# Correlation of Chest Wall Separation and Central Lung Distance With Ipsilateral Lung Dose in Post-operative Breast Cancer Patients Treated With Intensity-Modulated Radiotherapy (IMRT): A Retrospective Study

**DOI:** 10.7759/cureus.111236

**Published:** 2026-06-21

**Authors:** Yamini Bisht, Shashank Shekhar, Vishwadeep Mishra, Anjalikrishna NP

**Affiliations:** 1 Department of Radiotherapy, All India Institute of Medical Sciences Gorakhpur, Gorakhpur, IND

**Keywords:** breast cancer, central lung distance, chest wall separation, hypofractionation, intensity-modulated radiotherapy, lung dose–volume parameters

## Abstract

Objective

Post-operative chest wall radiotherapy with or without regional nodal irradiation is a cornerstone of breast cancer management. With increasing adoption of hypofractionated intensity-modulated radiotherapy (IMRT), minimizing incidental lung irradiation remains important despite improvements in dose conformity. Conventional geometric surrogates such as central lung distance (CLD) and chest wall separation (CWS), widely used in two-dimensional (2D) and three-dimensional (3D) conformal radiotherapy, have been less extensively evaluated in the IMRT era. This study assessed the relationship between these geometric parameters and ipsilateral lung dose in post-operative breast cancer patients treated with hypofractionated IMRT.

Methods

Thirty-six post-operative breast cancer patients treated at the Department of Radiotherapy, All India Institute of Medical Sciences (AIIMS), Gorakhpur, India, were retrospectively analysed. Twenty-eight patients (77.8%) had right-sided disease and eight (22.2%) had left-sided disease. All patients received hypofractionated IMRT to the chest wall with or without supraclavicular fossa irradiation to a dose of 40 Gy in 15 fractions. CWS was defined as the tangential distance between the anterior and posterior extents of the contoured chest wall on axial CT images, averaged across three central slices. CLD was measured as the perpendicular distance from the posterior chest wall into the ipsilateral lung. Dosimetric parameters analysed included ipsilateral lung V5, V10, V20, and mean lung dose (MLD). Pearson correlation analysis was used to evaluate associations between geometric and dosimetric parameters. Patients were additionally stratified into two groups based on CWS (<16 cm and ≥16 cm), and intergroup comparisons were performed using a two-tailed independent samples t-test.

Results

CLD demonstrated significant positive correlations with all evaluated ipsilateral lung dose parameters, with increasing correlation strength from low-dose to higher-dose metrics (percentage lung volume receiving ≥5 Gy (V5): r = 0.445, p = 0.006; percentage lung volume receiving ≥10 Gy (V10): r = 0.500, p = 0.002; percentage lung volume receiving ≥20 Gy (V20): r = 0.579, p < 0.001; MLD: r = 0.592, p < 0.001). CWS showed weaker inverse correlations with lung dose parameters, reaching statistical significance only for lung V20 (r = −0.418, p = 0.011). On subgroup analysis, patients with CWS <16 cm demonstrated significantly higher lung V20 values compared with those with CWS ≥16 cm (29.01 ± 3.13% vs 26.83 ± 0.92%; t = 2.964, p = 0.005). MLD was also higher in the CWS <16 cm group (14.92 ± 0.94 Gy vs 14.13 ± 1.34 Gy), although this difference did not reach statistical significance (t = 1.989, p = 0.055).

Conclusion

CLD is a significant geometric predictor of ipsilateral lung dose in post-operative breast cancer patients treated with hypofractionated IMRT. Significant associations were observed between CLD and all evaluated lung dose-volume parameters, particularly lung V20 and MLD. CWS demonstrated a weaker but significant inverse association with lung V20. These findings highlight the continued relevance of simple anatomical surrogates in contemporary breast radiotherapy planning and suggest that CLD may serve as a practical indicator of increased lung dose exposure during treatment planning.

## Introduction

Why lung dose still matters in the intensity-modulated radiotherapy (IMRT) era

Adjuvant radiotherapy remains a cornerstone in the management of breast cancer, significantly reducing locoregional recurrence and improving survival following surgery. However, incidental irradiation of the ipsilateral lung continues to carry the risk of radiation pneumonitis (RP) and long-term pulmonary sequelae, even with modern techniques such as IMRT. Historically, clinicians recognized that larger volumes of lung receiving intermediate doses (e.g., percentage lung volume receiving ≥20 Gy (V20)-percentage lung volume receiving ≥30 Gy (V30)) were associated with increased radiological and clinical lung changes. Dose-volume constraints aimed at reducing lung V20 have been correlated with lower rates of radiological pneumonitis after breast radiotherapy, underscoring the importance of limiting the volume of lung receiving ≥20 Gy [1].

Despite the intuitive advantages of IMRT for conformality and organ-at-risk (OAR) sparing, patient anatomy still influences dose distribution. Lee et al. (2020) and Park et al. (2021) demonstrated that lung dose-volume metrics (percentage lung volume receiving ≥5 Gy (V5), percentage lung volume receiving ≥10 Gy (V10), V20, and mean lung dose (MLD) are predictive of pulmonary radiation exposure and toxicity risk [2,3].

Adoption of hypofractionated schedules like 40 Gy in 15 fractions (40 Gy/15#) (five times a week) to post-modified radical mastectomy chest wall has become increasingly widespread due to convenience, cost-effectiveness, and radiobiological equivalence to conventional fractionation in breast tissue. The START Trial B investigators (2008) demonstrated that hypofractionated breast radiotherapy delivered as 40 Gy in 15 fractions achieved equivalent tumor control compared with conventional fractionation, and Haviland et al. (2013) subsequently confirmed the durability of these outcomes with lower late normal tissue toxicity at 10-year follow-up [4,5]. Lee et al. (2020) established that hypofractionated breast radiotherapy using modern techniques is associated with a lower incidence of RP compared with conventional schedules [2]. Nonetheless, the radiobiological implications of higher dose per fraction on lung tissue remain an area of active study, as the interplay between fraction size and lung tolerance is complex and influenced by dose distribution metrics. 

Geometry as an underappreciated determinant

In early radiotherapy practice, geometric surrogates like central lung distance (CLD) and chest wall separation (CWS) were widely used in two-dimensional (2D) planning to estimate the extent of lung irradiated in breast fields. CLD was defined as the distance from the posterior field edge to the chest wall and served as a simple predictor of the portion of lung encompassed by tangential beams. CWS provided a surrogate measure of anteroposterior patient build and thoracic geometry, which influenced field penetration and scatter dose [6].

With the advent of three-dimensional conformal radiotherapy (3D-CRT) and subsequently IMRT, planning shifted to volumetric dose-volume histogram (DVH) analysis, offering more precise organ dose quantification. However, geometric parameters continue to carry diagnostic and predictive value, particularly in resource-constrained settings or during initial plan assessment. Das et al. (2013) and Adeneye et al. (2021) demonstrated that geometric surrogates such as CLD and CWS correlate with lung and heart dose-volume parameters in breast radiotherapy [6,7].

Despite this, there remains a relative paucity of data from contemporary IMRT cohorts, especially within the Indian context, where patterns of delivery, patient body habitus, and resource utilization may differ from Western populations. This underscores the need to re-evaluate whether traditional geometric surrogates retain predictive value in modern planning paradigms.

Why this study was necessary

Several factors motivated this retrospective investigation. First, Indian institutional datasets on hypofractionated IMRT for post-operative breast cancer are limited, particularly with respect to lung dosimetry and geometry. Validation of classical predictors such as CLD and CWS in the IMRT context has important implications for planning quality assurance and toxicity risk stratification in routine practice.

Second, DVH-based dosimetric parameters like V5, V10, V20, and MLD offer quantitative insight into lung exposure but are inherently descriptive of the plan rather than the anatomy that shaped it. Understanding how patient-specific anatomical measures relate to these DVH metrics may help clinicians anticipate difficult cases, tailor optimization strategies, and potentially adopt geometry-based thresholds during plan review.

Finally, while clinical toxicity outcomes such as symptomatic RP are ideally measured prospectively, the small sample size and retrospective nature of many institutional series preclude robust clinical correlation. Focusing on geometric and dosimetric associations provides a valuable intermediate step, enhancing our understanding of dose prediction and anatomical influence in breast IMRT, and setting the stage for future larger studies linking anatomy, dose, and clinical outcomes.

## Materials and methods

Study design and ethical framework

This study was conducted as a retrospective dosimetric analysis of post-operative breast cancer patients treated with adjuvant radiotherapy at the Department of Radiotherapy, All India Institute of Medical Sciences (AIIMS), Gorakhpur, India. Treatment plans and planning CT datasets were reviewed retrospectively to evaluate the relationship between chest wall geometry and ipsilateral lung dose-volume parameters.

The Institutional Human Ethics Committee of AIIMS Gorakhpur issued approval (IHEC/AIIMS-GKP/BMR/749/2026). As this was a retrospective analysis of anonymized treatment planning data with no direct patient intervention, the study was performed in accordance with institutional ethical standards and the principles of the Declaration of Helsinki. 

Patient population

A total of 36 consecutive post-operative non-metastatic breast cancer patients treated with IMRT were included in the analysis. Of these, 28 patients (77.8%) had right-sided breast cancer, and eight patients (22.2%) had left-sided disease. Laterality was not stratified because the study focused on ipsilateral lung dose parameters.

All patients had undergone definitive surgery and subsequently received adjuvant radiotherapy to the chest wall with or without supraclavicular fossa irradiation, based on clinical and pathological risk factors. Patients receiving hypofractionated IMRT to a total dose of 40 Gy in 15 fractions were eligible for inclusion. No patients were excluded on the basis of age, laterality, or systemic therapy.

Simulation and contouring

All patients underwent CT simulation in the supine position on a breast board with both arms abducted above the head. Axial CT images were acquired with an appropriate slice thickness suitable for three-dimensional treatment planning.

Target volumes and OAR were contoured on the planning CT datasets. The chest wall clinical target volume (CTV) was delineated in accordance with Radiation Therapy Oncology Group (RTOG) breast contouring guidelines, incorporating the chest wall musculature and surgical bed as appropriate. The ipsilateral lung was contoured as a standard OAR, excluding the trachea and main bronchi, following accepted thoracic contouring conventions [8].

Use of standardized contouring guidelines was intended to minimize inter-observer variability and ensure consistency across all evaluated plans.

Treatment planning

All patients were treated using an IMRT technique. Treatment planning was performed on a commercial treatment planning system, with beam arrangements optimized to achieve adequate target coverage while minimizing dose to OAR.

The prescribed dose was 40 Gy delivered in 15 fractions to the chest wall, consistent with commonly adopted hypofractionated breast radiotherapy regimens. Dose calculations were performed using the institution’s standard dose calculation algorithm (collapsed cone/Anisotropic Analytical Algorithm (AAA)/equivalent, as per system availability), and DVHs were generated for all targets and OAR.

Plan evaluation prioritized adequate chest wall and nodal coverage while maintaining ipsilateral lung doses within clinically acceptable limits.

Definition of geometric parameters

Assessment of geometric parameters constituted a central component of this study.

Chest Wall Separation

CWS was defined as the tangential distance between the anterior and posterior extents of the contoured chest wall target volume on axial CT images. Measurements were performed on three central axial slices through the chest wall to account for anatomical variability along the cranio-caudal axis. The mean of these three measurements was recorded as the representative CWS for each patient.

This averaging approach was adopted to reduce slice-specific bias and provide a more stable estimate of overall thoracic geometry.

**Figure 1:**
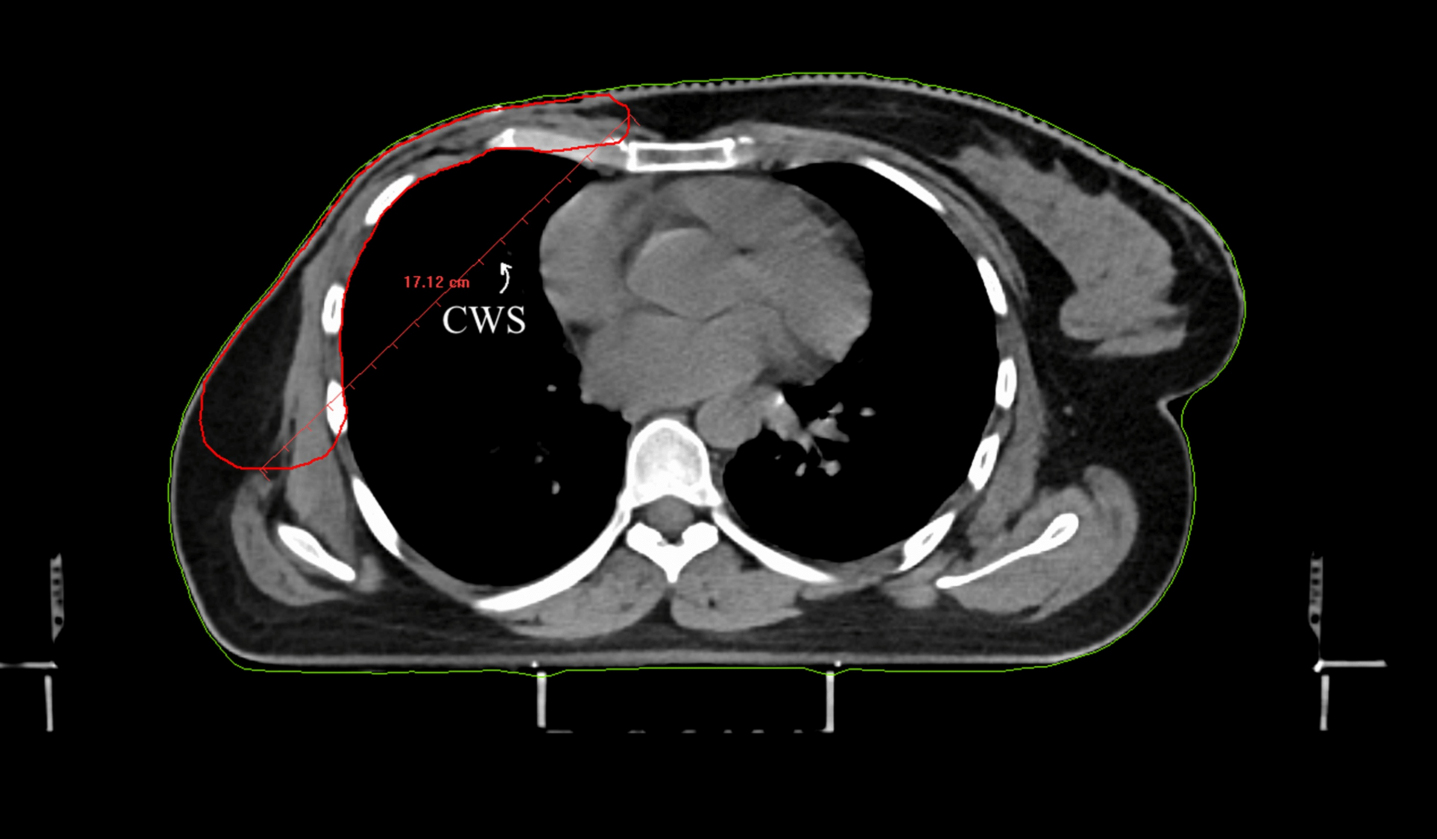
Axial planning CT image demonstrating the method used to measure chest wall separation (CWS). The contoured chest wall target volume is outlined (red). CWS is defined as the tangential distance between the anterior and posterior extents of the contoured chest wall on an axial slice, measured along the beam direction. The measurement shown represents one of three central slices used for averaging to obtain the final CWS value for each patient.

CWS was measured on axial planning CT images as illustrated in Figure 1.

Central Lung Distance

CLD is the distance of the lung included inside the tangential breast field, measured perpendicularly from the posterior edge of the field to the chest wall, at the center of the field on the portal image. In this study, the concept of CLD was adapted to CT-based planning, allowing direct anatomical measurement rather than reliance on simulator or portal images.

CLD was recorded on the axial slice corresponding to the central portion of the tangential field arrangement.

**Figure 2:**
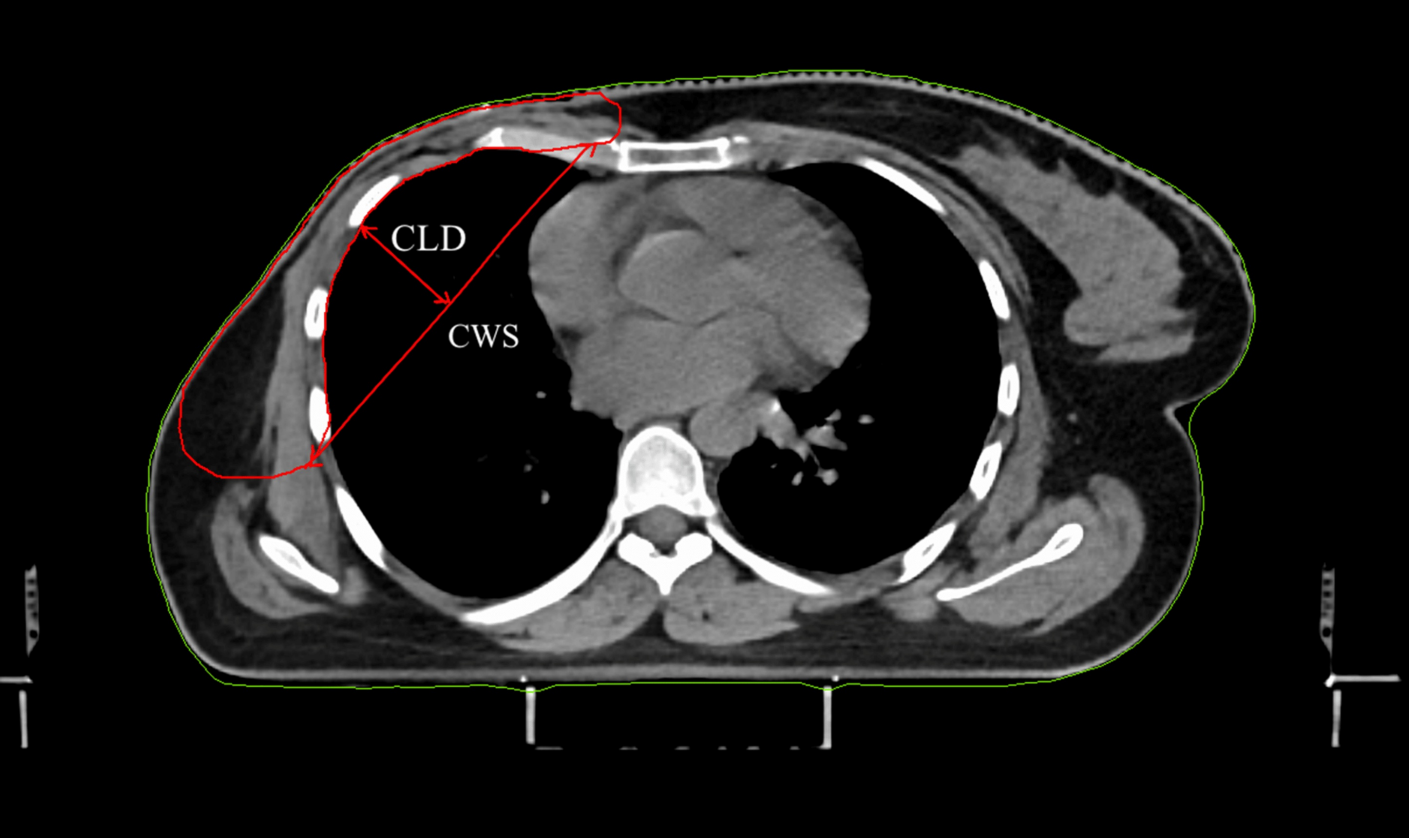
Axial planning CT image illustrating the geometric parameters analyzed in this study. Chest wall separation (CWS) is shown as the tangential distance between the anterior and posterior borders of the contoured chest wall target volume. Central lung distance (CLD) is measured as the perpendicular distance from the posterior edge of the chest wall target volume into the ipsilateral lung, representing the depth of the lung encompassed by the tangential beam arrangement.

The CT-based measurement of CLD and CWS is demonstrated in Figure 2.

Dosimetric parameters analysed

Ipsilateral lung dose-volume parameters extracted from the DVHs included V5, V10, V20, and MLD. These parameters were selected because of their established association with radiation-induced pulmonary toxicity and their frequent use in clinical practice and published literature [9].

Statistical analysis

The relationship between geometric parameters (CWS and CLD) and lung dosimetric indices (V5, V10, V20, and MLD) was assessed using Pearson correlation analysis. Pearson correlation coefficients (r) and corresponding p-values were reported.

For subgroup analysis, patients were stratified according to CWS into two groups: those with CWS <16 cm and those with CWS ≥16 cm. A CWS cutoff of 16 cm was chosen based on the institutional median value and prior dosimetric literature using similar thresholds.

Comparisons of lung V20 and MLD between these two groups were performed using a two-sample t-test assuming equal variances (two-tailed). A p-value <0.05 was considered statistically significant. Statistical analysis was performed using Microsoft Excel (Microsoft Corp., Redmond, WA, USA; with standard statistical formulas for Pearson correlation and t-tests).

## Results

Correlation between CLD and ipsilateral lung dose

**Figure 3:**
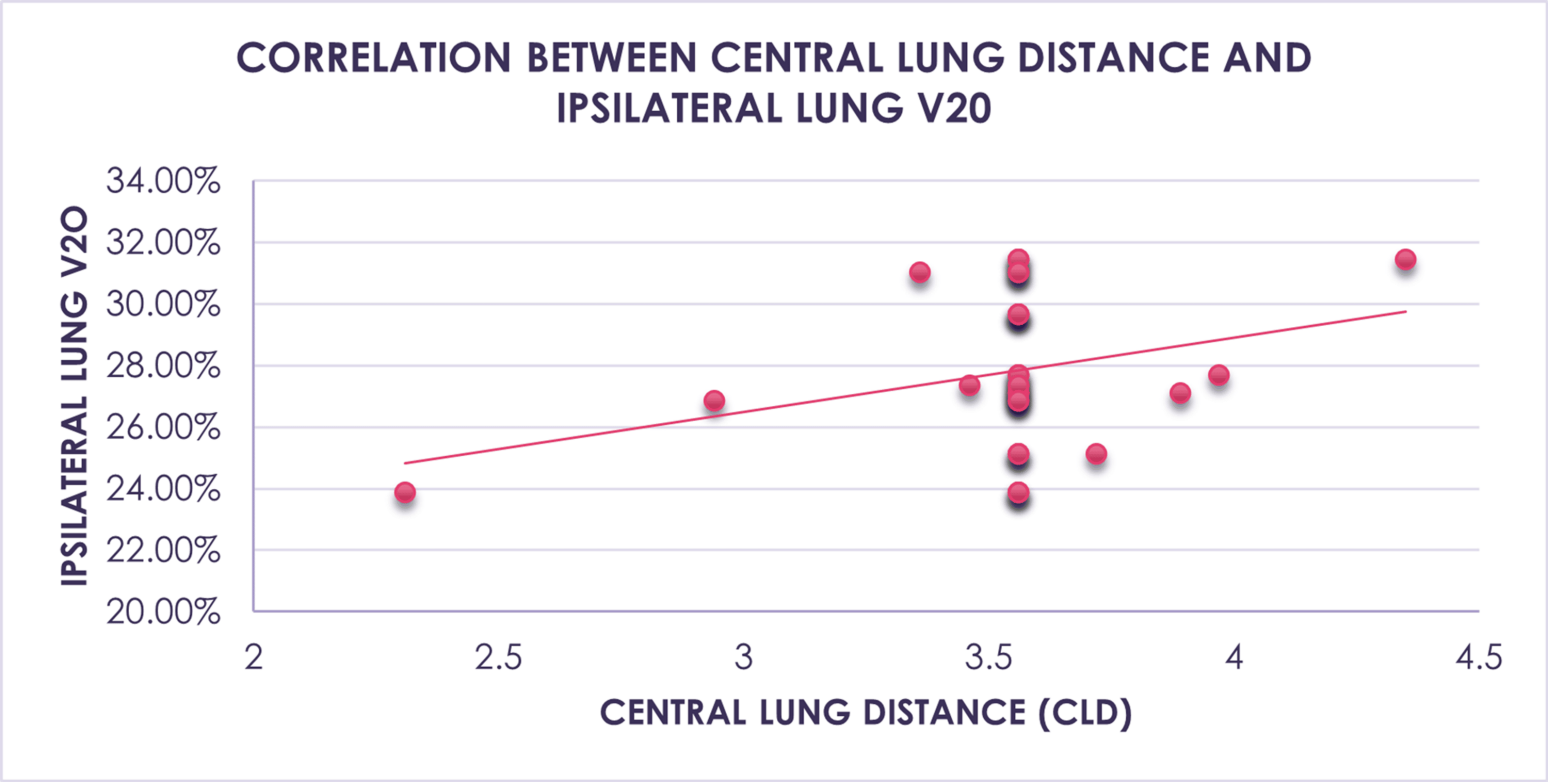
Scatter plot showing the correlation between central lung distance (CLD) and ipsilateral lung V20. Each point represents one patient (n = 36). The fitted regression line demonstrates a positive association between CLD and lung V20 (r = 0.579, p < 0.001). Statistical significance was defined as p < 0.05. Abbreviation: V20 = percentage lung volume receiving ≥20 Gy.

As shown in Figure 3 and Table 1, CLD demonstrated a statistically significant positive correlation with all evaluated ipsilateral lung dose-volume parameters. The strength of correlation increased progressively from low-dose to higher-dose metrics, suggesting a clinically relevant dose-geometry relationship.

**Table 1:** Pearson correlation vetween central lung distance (CLD) and ipsilateral lung dose parameters Abbreviations: V5 = percentage lung volume receiving ≥5 Gy; V10 = percentage lung volume receiving ≥10 Gy; V20 = percentage lung volume receiving ≥20 Gy. Data are presented as Pearson correlation coefficient (r) and corresponding p-values. Correlations were calculated using Pearson correlation analysis (n = 36). Statistical significance was defined as p < 0.05.

Lung Dosimetric Parameter	Pearson Correlation Coefficient (r)	p-value
Lung V5 (%)	0.445	0.006
Lung V10 (%)	0.500	0.002
Lung V20 (%)	0.579	<0.001
Mean Lung Dose (Gy)	0.592	<0.001

A moderate positive correlation was observed between CLD and lung V5 (r = 0.445, p = 0.006) as well as lung V10 (r = 0.500, p = 0.002). Stronger correlations were identified for higher-dose parameters, with CLD showing its strongest association with lung V20 (r = 0.579, p < 0.001) and MLD (r = 0.592, p < 0.001).

These findings indicate that increasing CLD is associated with greater ipsilateral lung irradiation and suggest that posterior thoracic geometry remains an important determinant of lung dose even in IMRT-based treatment planning.

CWS and lung dose: a weaker relationship

CWS demonstrated inverse correlations with ipsilateral lung dose-volume parameters, as shown in Table 2. Weak negative correlations were observed between CWS and lung V5 (r = −0.103, p = 0.550) and lung V10 (r = −0.106, p = 0.540), which were not statistically significant.

**Table 2:** Pearson correlation between chest wall separation (CWS) and ipsilateral lung dose parameters Abbreviations: V5 = percentage lung volume receiving ≥5 Gy; V10 = percentage lung volume receiving ≥10 Gy; V20 = percentage lung volume receiving ≥20 Gy. Data are presented as Pearson correlation coefficient (r) and corresponding p-values. Correlations were calculated using Pearson correlation analysis (n = 36). Statistical significance was defined as p < 0.05.

Lung Dosimetric Parameter	Pearson Correlation Coefficient (r)	p-value
Lung V5 (%)	-0.103	0.550
Lung V10 (%)	-0.106	0.540
Lung V20 (%)	-0.418	0.011
Mean Lung Dose (Gy)	-0.266	0.117

A moderate negative correlation was observed between CWS and lung V20 (r = −0.418, p = 0.011), indicating that patients with larger CWS tended to receive lower volumes of lung irradiation at clinically relevant dose levels. The correlation between CWS and MLD was weak and did not achieve statistical significance (r = −0.266, p = 0.117).

These findings suggest that although CWS is less strongly associated with lung dose than CLD, it may still influence intermediate-dose lung exposure in patients treated with IMRT.

Subgroup analysis by CWS

Patients were stratified into two groups according to CWS: CWS <16 cm (n = 16, 44.4%) and CWS ≥16 cm (n = 20, 55.6%). As shown in Table 3, patients with smaller CWS demonstrated higher ipsilateral lung dose parameters compared with those with larger CWS.

**Table 3:** Comparison of ipsilateral lung dose parameters by CWS group Abbreviations: CWS = chest wall separation; SD = standard deviation. Continuous variables are presented as Mean ± SD. Group comparisons were performed using a two-tailed independent samples t-test. Statistical significance was defined as p < 0.05.

Parameter	CWS <16 cm (n = 16) Mean ± SD	CWS ≥16 cm (n = 20) Mean ± SD	t-value	p-value
Lung V20 (%)	29.01 ± 3.13	26.83 ± 0.92	2.964	0.005
Mean Lung Dose (Gy)	14.92 ± 0.94	14.13 ± 1.34	1.989	0.055

The mean lung V20 was significantly higher in the CWS <16 cm group (29.01 ± 3.13%) than in the CWS ≥16 cm group (26.83 ± 0.92%) (t = 2.964, p = 0.005). Similarly, the MLD was higher among patients with smaller CWS (14.92 ± 0.94 Gy) compared with those with larger CWS (14.13 ± 1.34 Gy); however, this difference did not reach statistical significance (t = 1.989, p = 0.055).

These findings indicate that patients with smaller CWS are more likely to receive higher ipsilateral lung doses, particularly with respect to lung V20, which demonstrated a statistically significant difference between groups.

Summary of key findings

CLD demonstrated statistically significant positive correlations with all evaluated ipsilateral lung dose parameters, with the strongest associations observed for lung V20 and MLD. CWS showed inverse correlations with lung dose metrics and was significantly associated with lung V20. Subgroup analysis further demonstrated significantly higher lung V20 values among patients with CWS <16 cm, while MLD showed a similar trend that did not reach statistical significance. Collectively, these findings support the continued relevance of geometric parameters, particularly CLD, as predictors of ipsilateral lung dose in post-operative breast IMRT.

## Discussion

This retrospective dosimetric analysis evaluated the relationship between two geometric parameters, CLD and CWS, and ipsilateral lung dose in post-operative breast cancer patients treated with hypofractionated IMRT. Our findings demonstrate that CLD is significantly associated with all evaluated lung dose-volume parameters, while CWS shows a significant inverse association with lung V20 and influences lung dose exposure on subgroup analysis. These results suggest that anatomical geometry continues to play an important role in determining lung irradiation despite advances in treatment planning and dose modulation.

Clinical relevance of CLD

Among the evaluated geometric parameters, CLD emerged as the strongest predictor of ipsilateral lung dose. Significant positive correlations were observed between CLD and all dose-volume metrics, with the strongest associations noted for lung V20 (r = 0.579, p < 0.001) and MLD (r = 0.592, p < 0.001). The progressive increase in correlation strength from V5 to V20 suggests that posterior thoracic geometry exerts a greater influence on clinically relevant intermediate-dose lung exposure than on low-dose irradiation.

Our findings are consistent with those of Chen et al. [10], who demonstrated that CT-based anatomical indices, including CLD, can predict ipsilateral lung dose prior to treatment planning. Similarly, Das et al. [6] reported significant correlations between geometric breast parameters and lung dose-volume metrics in breast radiotherapy. Collectively, these observations support the concept that patient anatomy remains an important determinant of dosimetry even when advanced planning techniques are employed. Adeneye et al. [7] further reported that although IMRT improves dose homogeneity and target coverage, anatomical factors continue to influence OAR doses, supporting the relevance of geometric predictors in contemporary breast radiotherapy planning.

CWS and lung dose

In contrast to CLD, CWS demonstrated weaker associations with lung dose parameters. While correlations with V5, V10, and MLD were not statistically significant, a moderate inverse correlation was observed between CWS and lung V20 (r = −0.418, p = 0.011). Furthermore, subgroup analysis revealed significantly higher lung V20 values among patients with CWS <16 cm compared with those with CWS ≥16 cm (29.01% vs 26.83%, p = 0.005).

These findings suggest that patients with smaller thoracic dimensions may experience greater lung irradiation within clinically relevant dose ranges. The inverse relationship observed in this study differs somewhat from historical observations in conventional tangential radiotherapy, where larger separation often resulted in greater normal tissue exposure. The discrepancy likely reflects the ability of IMRT optimization to compensate for variations in anterior chest wall thickness, thereby reducing the dosimetric influence of CWS compared with older planning techniques [7,11].

Although CWS appears less predictive than CLD, the significant association with lung V20 indicates that it may still provide useful anatomical information during treatment planning and plan review.

Implications for hypofractionated breast IMRT

All patients in the present study received hypofractionated radiotherapy to a total dose of 40 Gy in 15 fractions, a regimen supported by the START Trial [4] and long-term follow-up data reported by Haviland et al. [5], and widely adopted in contemporary breast cancer practice. While hypofractionation has demonstrated excellent tumor control and acceptable toxicity, minimizing lung exposure remains important because RP and long-term pulmonary changes continue to correlate with dose-volume parameters such as V20 and MLD [9,12].

The observed relationship between CLD and these established dosimetric predictors suggests that simple anatomical measurements may assist clinicians in identifying patients at increased risk of lung dose escalation before detailed optimization is performed. Such information may facilitate early implementation of lung-sparing strategies and improve planning efficiency.

Comparison with previous literature

Several investigators have reported associations between geometric parameters and OAR doses in breast radiotherapy. Das et al. [6] demonstrated correlations between geometric breast measurements and lung dose-volume parameters in conventional treatment techniques. Chen et al. [10] subsequently showed that CT-based anatomical predictors remain useful in contemporary planning environments. Our study extends these observations to a cohort treated exclusively with hypofractionated IMRT and confirms that geometric predictors remain clinically relevant despite advances in treatment delivery.

The stronger predictive performance of CLD compared with CWS observed in our analysis further supports the notion that posterior thoracic geometry more directly determines lung inclusion within treatment fields than anterior chest wall dimensions. Hall [13] highlighted that although IMRT improves target conformity, it may increase the volume of normal tissues receiving low-dose radiation because of the use of multiple beam angles and higher monitor units.

Indian context and practical implications

In many resource-constrained environments, sophisticated motion-management techniques such as deep inspiration breath hold may not be universally available. Under such circumstances, readily obtainable geometric parameters can provide valuable supplementary information during treatment planning. The significant association between CLD and all evaluated lung dose metrics suggests that CLD may serve as a practical and inexpensive planning aid, particularly in centres where rapid risk assessment is required.

The findings also contribute locally generated evidence from an Indian cohort, addressing an area where published dosimetric data remain relatively limited. Furthermore, variability in the adoption of dose constraints and planning approaches across institutions has been reported, highlighting the value of simple and reproducible anatomical predictors that can be applied consistently in routine clinical practice [14].

Limitations

This study has several limitations. The retrospective design limits the ability to establish causality and precludes assessment of clinical outcomes such as symptomatic RP. The sample size was relatively modest, although statistically significant associations were identified despite this limitation. In addition, left-sided and right-sided breast cancers were analysed together because the primary endpoint was ipsilateral lung dose rather than cardiac exposure. Finally, the study evaluated dosimetric endpoints alone and did not incorporate respiratory motion management, clinical toxicity data, or long-term follow-up outcomes.

Future directions

What Planners Can Take Away Today

The findings of the present study suggest that CLD remains a clinically relevant geometric predictor of ipsilateral lung dose even in the era of IMRT. Significant positive correlations were observed between CLD and all evaluated lung dose-volume parameters, with the strongest associations identified for lung V20 and MLD. Consequently, early recognition of patients with high CLD during treatment planning may help identify cases at increased risk of lung dose escalation. Such patients may benefit from additional optimization strategies, including modification of beam arrangements, adjustment of segment weighting, or the incorporation of advanced planning techniques aimed at reducing lung exposure. Although CWS demonstrated weaker associations with lung dose parameters, its significant inverse relationship with lung V20 suggests that it may still provide useful supplementary anatomical information, particularly in resource-constrained settings where rapid geometric assessment can assist plan evaluation and quality assurance.

What Future Studies Should Address

Further research is warranted to validate and expand upon these findings. Larger multicentric studies with more diverse patient populations are needed to establish robust geometric thresholds that can reliably predict lung dose exposure during breast radiotherapy. Incorporation of clinical endpoints, including radiographic and symptomatic RP, would facilitate direct correlation between dosimetric predictors and patient outcomes. Future investigations should also evaluate left-sided and right-sided breast cancers separately, given the additional considerations of cardiac irradiation in left-sided disease. Moreover, the influence of contemporary techniques such as deep inspiration breath hold, respiratory gating, adaptive radiotherapy, and proton therapy on the relationship between patient geometry and lung dose should be explored. Such studies may help refine individualized treatment planning approaches and further optimize OAR sparing in breast cancer radiotherapy.

## Conclusions

This retrospective dosimetric analysis demonstrates that CLD is a significant geometric predictor of ipsilateral lung dose in post-operative breast cancer patients treated with hypofractionated IMRT. Significant positive correlations were observed between CLD and all evaluated lung dose-volume parameters, with the strongest associations identified for lung V20 and MLD. These findings indicate that increasing CLD is associated with greater lung irradiation and highlight the continued importance of posterior thoracic geometry in contemporary breast radiotherapy planning. CWS showed a weaker overall relationship with lung dose; however, a significant inverse correlation was observed with lung V20, and patients with CWS <16 cm demonstrated significantly higher V20 values than those with larger CWS. These results suggest that chest wall geometry may also influence clinically relevant lung exposure, although to a lesser extent than CLD.

Collectively, the findings reinforce the continued relevance of simple anatomical surrogates in the IMRT era. Geometric parameters such as CLD can serve as practical and readily available indicators of potential lung dose escalation, facilitating early identification of higher-risk cases and supporting optimization of treatment planning. Larger prospective studies incorporating clinical pulmonary toxicity outcomes are warranted to further validate these observations and establish clinically useful geometric thresholds for routine practice.
